# Temperature and Emissivity Inversion Accuracy of Spectral Parameter Changes and Noise of Hyperspectral Thermal Infrared Imaging Spectrometers

**DOI:** 10.3390/s20072109

**Published:** 2020-04-08

**Authors:** Honglan Shao, Chengyu Liu, Chunlai Li, Jianyu Wang, Feng Xie

**Affiliations:** 1 Key Lab of Space Active Opto-Electronic Techniques, Shanghai Institute of Technical Physics, Chinese Academy of Sciences, Shanghai 200083, China; shaoko@163.com (H.S.); liuchengyu@mail.sitp.ac.cn (C.L.); lichunlai@mail.sitp.ac.cn (C.L.); xf@mail.sitp.ac.cn (F.X.); 2 University of Chinese Academy of Sciences, Beijing 100049, China

**Keywords:** hyperspectral thermal infrared, temperature and emissivity separation, inversion error, center wavelength shift, FWHM change, instrument noise

## Abstract

The emergence of hyperspectral thermal infrared imaging spectrometers makes it possible to retrieve both the land surface temperature (LST) and the land surface emissivity (LSE) simultaneously. However, few articles focus on the problem of how the instrument’s spectral parameters and instrument noise level affect the LST and LSE inversion errors. In terms of instrument development, this article simulated three groups of hyperspectral thermal infrared data with three common spectral parameters and each group of data includes tens of millions of simulated radiances of 1525 emissivity curves with 17 center wavelength shift ratios, 6 full width at half maximum (FWHM) change ratios and 6 noise equivalent differential temperatures (NEDTs) under 15 atmospheric conditions with 6 object temperatures, inverted them by two temperature and emissivity separation methods (ISSTES and ARTEMISS), and analyzed quantitatively the effects of the spectral parameters change and noise of an instrument on the LST and LSE inversion errors. The results show that: (1) center wavelength shifts and noise affect the inversion errors strongly, while FWHM changes affect them weakly; (2) the LST and LSE inversion errors increase with the center wavelength shift ratio in a quadratic function and increase with FWHM change ratio slowly and linearly for both the inversion methods, however they increase with NEDT in an S-curve for ISSTES while they increase with NEDT slightly and linearly for ARTEMISS. During the design and development of a hyperspectral thermal infrared instrument, it is highly recommended to keep the potential center wavelength shift within 1 band and keep NEDT within 0.1K (corresponding LST error < 1K and LSE error < 0.015) for normal applications and within 0.03K (corresponding LST error < 0.5K and LSE error < 0.01) for better application effect and level.

## 1. Introduction

The land surface temperature (LST) and land surface emissivity (LSE) are key parameters for many areas such as mineral identification [1,2], gas plume detection [3], plant species [4], soil moisture retrieval [5]. The emergence of many hyperspectral thermal infrared (HTIR) imaging spectrometers, which have hundreds of contiguous spectral bands in the atmospheric window of 8–14 μm with spectral resolution higher than 100 nm, especially the commercial ones, has helped move thermal infrared hyperspectral remote sensing from concept to reality. We can retrieve simultaneously the LST and LSE of one pixel from the HTIR data with a temperature and emissivity separation (TES) algorithm. With the development of thermal infrared technology, many HTIR imaging spectrometers have appeared, for example, SEBASS [6], MAKO [7], Hyper-Cam LW [8], Sieleters B3 [9], hyperspectral thermal emission spectrometer (HyTES) [10], AisaOWL [11] and the airborne thermal infrared hyperspectral imaging system (ATHIS) [12].

Different from single-band, dual-band or multispectral thermal infrared remote sensing which focus on the retrieval of LST, HTIR remote sensing is able to retrieve the LST and LSE simultaneously with higher quantitative inversion accuracy, which a way for an important application mode of HTIR data, namely using the retrieval LSE spectrum to identify ground objects. The spectral information in the LSE reflects the pattern characteristics of an object, which can also be understood as the changing characteristics of the emissivity curve. However, most ground objects have high emissivity and their emissivity changes are relatively small (for example, the average emissivity of water is about 0.98, and its fluctuation is only 0.02.) Such small changes are general characteristics of low-reflection ground objects in reflective optical remote sensing. On the one hand, it naturally comes to mind to improve the performance of the HTIR instrument when we want to obtain high-quality emissivity curves from HTIR data to describe the emission features of ground objects, or even to build connection between the emissivity curve of an object and its physical properties. On the other, from the principle of thermal infrared imaging, it is known that the spectral response accuracy and noise level of the instrument directly affect the quality of the acquired data and further affect HTIR inversion results. Therefore, it is necessary to study the quantitative influence of spectral response change (namely center wavelength shift and FWHM change) and instrument noise level on the LST and LSE inversion, which will provide significant support for the instrument design and development and the HTIR applications.

The difficulty of inversing the LST and LSE simultaneously from the hyperspectral or multispectral thermal infrared data observed in the same phase, namely separating the temperature and emissivity, lies in solving *N* equations (one sensor output radiance spectrum with *N* bands) to get *N* + 1 unknowns (*N* emissivities and one temperature), which is an underdetermined equation problem. It is necessary to introduce additional conditions in order to solve the problem, usually increasing the number of equations by proposing assumptions about the target emissivity spectrum shape or target temperature. There are several existing TES algorithms provided for multispectral thermal infrared data, such as reference band method (MBR) [13], normalized emissivity method [14,15], alpha residues method [16], temperature-independent spectral indices (TISI) [17], spectral ratio method [18], maximum-minimum difference (MMD) [19], split-window [20], or grey body emissivity [21]. Compared with single-band or multispectral thermal infrared data, the hyperspectral thermal infrared data can provide more hypothetical conditions in line with real physical properties of objects for TES, effectively increasing the stability of the underdetermined equations to be solved. Most of existing TES algorithms for hyperspectral thermal infrared data are based on the well-known observation that the emissivity spectra of most materials are much smoother than the atmospheric transmission features (smoothness assumption). Based on cost function representations, there are spectral smoothing index class algorithms, e.g., the iterative spectrally smooth temperature-emissivity separation (ISSTES) [22] and its improved version, the automatic retrieval of temperature and emissivity using spectral smoothness (ARTEMISS) [23]; downwelling radiance residual class algorithms, e.g., the downwelling radiance residual index (DRRI) [24], the correlation-based temperature and emissivity separation (CBTES) [25], stepwise refining algorithm of temperature and emissivity separation (SRATES) [26]; linear constraint class, e.g., linear spectral emissivity constraint (LSEC) [27], improved LSEC (I-LSEC) [28], pre-estimate shape LSEC (PES-LSEC) [29]; and wavelet class algorithms, e.g., wavelet transform method for separating temperature and emissivity (WTTES) [30], multi-scale wavelet-based temperature and emissivity separation (MSWTES)[31].

As many TES more algorithms are described, more articles are devoted to the sensitivity analysis of TES algorithms. The impact factors of a TES algorithm performance are: assumptions reasonableness, robustness, atmospheric correction accuracy, spectral calibration accuracy, instrument noise and others [32,33,34,35,36]. Among the literature [33] and [36] involve the influence analysis of spectral calibration accuracy and instrument noise on the TES algorithms. In [33], an error analysis for the ARTEMISS algorithm shows that spectral shifts of more than 1/20th of the spectral sampling can produce fitting errors of the order of the sensor noise and errors in the FWHM of up to 10% still yield useful results. In [36], the researchers studied the influence of the instrument spectral properties (the center wavenumber shifts were 4%, 20%, 40%, 60% and FWHM were widen by 5%, 10%, 20%) and noise (Gaussian random, 0.1 K, 0.2 K) on the retrieval results for five TES algorithms, and the results shows that even if some spectral response changes cause only a slight change in the observed radiance or the bright temperature spectral data they have a great impact on the LST and LSE retrieval. The above studies both have shown that the changes in spectral response characteristics have a great impact on the TES inversion results, however these related studies have paid more attention to exploring the application boundary conditions of the newly proposed TES algorithm or to prove the performance of the new method, moreover the number of samples are small, for example 54 in [36], and the emissivity values of the samples in their experiments are high. Although TES algorithm improvements are helpful to improve the application of HTIR data under certain conditions, the advancement in the manufacturing level of HTIR instruments and quantification level of HTIR data can better promote the development of HTIR remote sensing from the source. Therefore, from the perspective of instrument manufacturing, we analyzed quantitatively the influence of the spectral parameters change and noise of an instrument on the TES inversion results, in order to provide a reference for HTIR instruments manufacturing and provide guidance on the application scope for a HTIR data with a given spectral parameters.

In terms of the instrument development, this article selected three instruments with currently common spectral resolutions (100 nm, 50 nm, and 35 nm) as representative examples, simulated tens of millions of radiances of 1525 emissivity curves with 17 center wavelength shift ratios, 6 FWHM change ratios and 6 noise equivalent differential temperatures (NEDTs) under 15 atmospheric conditions with 6 object temperatures, inverted them with two TES methods (ISSTES [22] and ARTEMISS [23]) respectively, and quantitatively analyzed the relationship between the LST and LSE inversion errors and each of the impact factors. After this Introduction section, Section 2 will introduce the data simulation process, the TES algorithms and the evaluation metrics for the inversion results, and Section 3, Section 4 and Section 5 will present the results, discussion and conclusions, respectively.

## 2. Data and Methods

**Figure 1:**
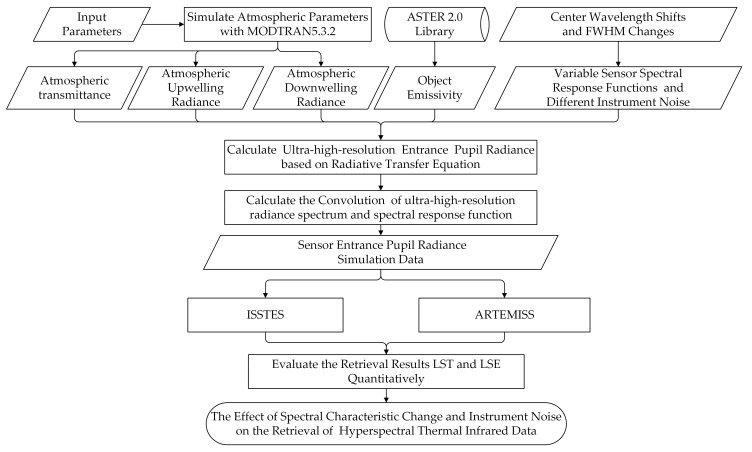
The flowchart of simulated at-sensor radiance generation, retrieval and results evaluation.

This study includes three experimental processes: data simulation, the LST and LSE inversion, and inversion results analysis. Figure 1 shows the flowchart. Firstly, three atmospheric parameters, namely atmospheric transmittance, atmospheric downwelling radiance and atmospheric upwelling radiance, are simulated using the MODTRAN 5.3.2 [37] software. The simulated sensor entrance pupil radiances with 1 nm spectral resolution are calculated with the simulated atmospheric parameters and the emissivity data calculated from ASTER 2.0 spectral library [38] based on the radiative transfer equation (RTE), then are convolved with the spectral response functions of three typical HTIR instruments and are added noise to obtain the noisy simulated sensor output radiances with corresponding spectral resolution, or called the simulated sensor radiances. Secondly, we use two TES algorithms (ISSTES and ARTEMISS) to invert the simulated sensor radiance data to obtain the LST and LSE, respectively. Finally, we evaluate the inversion results with its root mean square error (RMSE), analyze the relationships between the inversion errors and the center wavelength shift, FWHM change and NEDT and provide guidance for the development of new HTIR instruments.

### 2.1. Data Simulation

#### 2.1.1. Ultra-High-Resolution Sensor Entrance Pupil Radiance

The simulated entrance pupil radiance with ultra-high spectral resolution (1nm) is calculated according to the RTE. Here, the ultra-high-resolution is mainly relative to the wavelength of the thermal infrared band, because 1nm spectral resolution is ultra-high-resolution relative to the thermal infrared band. Ignoring multiple scattering, the RTE is:

(1) $$L_{obs}(\lambda_{m},T)=\left\{\varepsilon \left(\lambda_{m}\right)\cdot B(\lambda_{m},T)+\left[1-\varepsilon \left(\lambda_{m}\right)\right]\cdot L_{d}(\lambda_{m})\right\}\cdot \tau \left(\lambda_{m}\right)+L_{u}(\lambda_{m})$$

where, $L_{obs}(\lambda_{m},T)$ is the entrance pupil radiance at band *m* with the LST of *T*, *λ_m_* ∈ [7,14] μm, *λ_m−_*_1_–*λ_m_* = 1 nm, m=1,2,3,…,7001, $\varepsilon \left(\lambda_{m}\right)$ is the LSE, $B\left(\lambda_{m},T\right)$ is the blackbody radiance at *T*, $\tau \left(\lambda_{m}\right)$ is the atmospheric transmittance, $L_{d}(\lambda_{m})$ is the atmospheric downwelling radiance, and $L_{u}(\lambda_{m})$ is the atmospheric upwelling radiance.

According to the RTE, we can calculate the 1nm ultra-high-resolution sensor entrance pupil radiance $L_{obs}(\lambda_{m},T)$ of a certain ground object with temperature *T* under a certain atmospheric condition, if we know the ground object emissivity curve $\varepsilon \left(\lambda_{m}\right)$ and obtain three atmospheric parameters ($\tau \left(\lambda_{m}\right)$, $L_{d}(\lambda_{m})$ and $L_{u}(\lambda_{m})$) through simulation.

**Figure 2:**
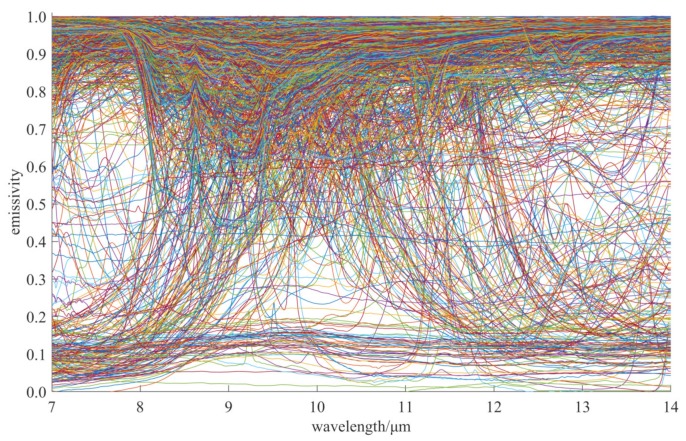
The 1525 emissivity curves from ASTER 2.0 Spectral Library.

Emissivity data $\varepsilon \left(\lambda_{m}\right)$ are from the ASTER 2.0 spectral library, commonly used for thermal infrared remote sensing research, which contains 2445 ground object reflectance spectra covering a rich variety of samples with different physical and chemical properties. In this study, we choose all the reflectance curves ranging from 7 to 14 μm in ASTER 2.0, for a total of 1525, consisting of 84 manmade materials, 60 stony meteorites, 910 minerals, 388 rocks, 75 soils, four types of vegetation, four water and five snow and ice. We obtain the object emissivity data from the reflectance data of ASTER 2.0 based on *ε* = 1 – *ρ* (*ρ* is the object reflectance) according to the principle of energy conservation and assuming the object transmittance is 0, then interpolate them to 1 nm spectral resolution. As shown in Figure 2, there are not only a large amount of emissivity curves with high value (>0.8), but also lots of emissivity curves with medium value (0.4–0.6) and low value (<0.2), as well as many fluctuating emissivity curves. These emissivity curves cover most scenarios in the real world.

The object temperature *T* is set according to the atmospheric temperature $T_{atom}$, $T=T_{atom}+\Delta T$, where ΔT is the temperature difference between the object and the atmosphere. If $T_{atom}>280K$, the value range of ΔT is [−5K, 20 K] with an interval of 5K; if $T_{atom}<280K$, the value range of ΔT is [−10K, 15K] with an interval of 5 K.

The atmospheric parameters ($\tau \left(\lambda_{m}\right)$, $L_{d}(\lambda_{m})$ and $L_{u}(\lambda_{m})$) are simulated by the MODTRAN 5.3.2 software. We get 15 groups of atmospheric parameters with five MODTRAN’s atmospheric models and three sensor altitudes, as shown in Table 1, while the other parameters of MODTRAN are the defaults.

#### 2.1.2. Sensor Output Radiance

In order to further simulate the noisy sensor radiance with its output spectral resolution, it is necessary to perform convolution on the simulated entrance pupil radiance with 1nm spectral resolution and the spectral response function of the HTIR imager and add noise. The sensor output radiance is simulated by

(2) $$L_{i}(\delta \lambda_{i},\delta \Delta \lambda_{i})=\frac{\int L_{obs}(\lambda )S_{i}(\lambda ,\lambda_{i},\Delta \lambda_{i},\delta \lambda_{i},\delta \Delta \lambda_{i})d\lambda }{\int S_{i}(\lambda ,\lambda_{i},\Delta \lambda_{i},\delta \lambda_{i},\delta \Delta \lambda_{i})d\lambda }+L\left(\eta \right)$$

where $L_{i}(\delta \lambda_{i},\delta \Delta \lambda_{i})$ is the sensor output radiance at the *i*-th band,$i\in \left[1,N\right]$, *N* is the number of bands; $L_{obs}(\lambda )$ is the ultra-high-resolution sensor entrance pupil radiance obtained in 2.1.1; $L\left(\eta \right)$ is the added noise, η is NEDT (the noise here refers to a comprehensive noise equivalent temperature difference, namely the random error of the data after calibration with no consideration of systematic calibration error); $S_{i}(\lambda ,\lambda_{i},\Delta \lambda_{i},\delta \lambda_{i},\delta \Delta \lambda_{i})$ is the spectral response function at the ith band, which is determined according to the adjusted center wavelength ($\lambda_{i}+\delta \lambda_{i}$) and FWHM ($\Delta \lambda_{i}+\delta \Delta \lambda_{i}$), where $\lambda_{i}$ and $\delta \lambda_{i}$ are the center wavelength and its shift respectively, while $\Delta \lambda_{i}$ and $\delta \Delta \lambda_{i}$ are the FWHM and its change respectively. The spectral response function is simulated using a Gaussian function as follows [39]:

(3) $$S_{i}(\lambda ,\lambda_{i},\Delta \lambda_{i},\delta \lambda_{i},\delta \Delta \lambda_{i})=\exp\left[-\frac{{\left(\lambda -\lambda_{i}-\delta \lambda_{i}\right)}^{2}}{{\left(\Delta \lambda_{i}+\delta \Delta \lambda_{i}\right)}^{2}/\ln16}\right]$$

In this study, three typical spectral resolutions (AisaOWL 100 nm, ATHIS 50 nm, HyTES 35 nm, details are shown in Table 2) were used to simulate the HTIR sensor spectral parameters and their different spectral response characteristics. The change in the spectral response function refers to the shift in the center wavelength and the change in FWHM. In this paper, there are 17 center wavelength shifts and six FWHM changes for each of the three sensors. The center wavelength shift $\delta \lambda_{i}$ is the product of the sensor spectral interval (half of the spectral resolution) and the center wavelength shift ratio. The center wavelength shift ratios are [0, ± 0.05, ± 0.1, ± 0.3, ± 0.5, ± 0.8, ± 1, ± 2, ± 3], where the positive sign indicates that the center wavelength shifts to the longer wavelength direction, reversely the negative sign indicates that the center wavelength shifts to the shorter wavelength direction. The FWHM change $\delta \Delta \lambda_{i}$ is the product of the spectral resolution and the FWHM change ratio. The FWHM change ratios are [0,0.1,0.2,0.3,0.4,0.5]. Generally, FWHM increases only, so $\delta \Delta \lambda_{i}$ is set to be positive. In addition, this study tested six noise levels which are characterized by the NEDT of [0, 0.1, 0.2, 0.3, 0.4, 0.5] K.

### 2.2. Temprature and Emissivity Separation Algorithms

The cost function is a key factor that affects the performance of the TES algorithms, whether it makes the underdetermined equations solvable by increasing constraints like a spectral iterative smoothing algorithm or by reducing the unknowns of the equations like a piecewise linear constraint method. Among the many TES algorithms mentioned in the Introduction, the standard deviation of the simulated sensor entrance pupil radiance and the true sensor entrance pupil radiance are most used as the cost functions. In the approximate processing of the emissivity curve, boxcar average is a more classic and commonly used processing method. Therefore, ISSTES and ARTEMISS, the two representative algorithms of the spectrum iterative smoothing family, are chosen in this study.

#### 2.2.1. ISSTES

The at-ground thermal infrared radiance includes not only the land surface’s own emitting radiance but also the atmospheric downwelling radiance reflected by the land surface. The basic idea of ISSTES is to establish a cost function based on such a fact that the emissivity spectrum curve of the ground is much smoother than the atmospheric downwelling radiance spectrum, then optimize the estimated temperature iteratively until the cost function is minimum where the land surface emissivity curve is optimally smoothed. The emissivity is calculated by:

(4) $$\varepsilon \left(\lambda \right)=\frac{L_{g}\left(\lambda ,T\right)-L_{d}(\lambda )}{B(\lambda ,T_{est})-L_{d}(\lambda )}=\frac{\left[L_{obs}(\lambda ,T)-L_{u}(\lambda )\right]/\tau \left(\lambda \right)-L_{d}(\lambda )}{B(\lambda ,T_{est})-L_{d}(\lambda )}$$

According to Equation (4), given a land surface temperature estimation, the corresponding emissivity spectrum can be calculated if the atmospheric parameters are known. The land surface temperature estimation needs to be accurate to eliminate the atmospheric absorption lines in the emissivity spectrum to get a relatively smooth emissivity spectrum, otherwise there will be residual atmospheric absorption lines in the estimated emissivity spectrum, making the emissivity spectrum rough. In order to evaluate the smoothness of the emissivity spectrum curve, a smoothness index $\sigma \left(\varepsilon \right)$ is considered as the cost function and defined as follows:

(5) $$\sigma \left(\varepsilon \right)=\mathrm{STDEV}\left[\varepsilon \left(\lambda_{i}\right)-\frac{\varepsilon \left(\lambda_{i-1}\right)+\varepsilon \left(\lambda_{i}\right)+\varepsilon \left(\lambda_{i+1}\right)}{3}\right],i=2,\ldots ,N-1$$

#### 2.2.2. ARTEMISS

Based on ISSTES’s idea of iterative spectral smoothing, ARTEMISS improves its iterative cost function. Instead of the smoothness index of emissivity spectrum curve, it calculates the corresponding sensor output radiance spectrum curve estimation according to the RTE and uses the standard deviation σ of the estimated sensor output radiance and the real sensor output radiance as the cost function. The estimated sensor output radiance is:

(6) $$L_{fit}\left(\lambda ,T_{est},{\bar{\varepsilon }}_{est}\right)=\tau \left(\lambda \right)\left[{\bar{\varepsilon }}_{est}\left(\lambda \right)B(\lambda ,T_{est})+\left(1-{\bar{\varepsilon }}_{est}\left(\lambda \right)\right)L_{d}(\lambda )\right]+L_{u}(\lambda )$$

The cost function is:

(7) $$\sigma \left(T_{est},{\bar{\varepsilon }}_{est}\right)=\sigma \left(L_{obj}\left(\lambda ,T\right)-L_{fit}\left(\lambda ,T_{est},{\bar{\varepsilon }}_{est}\right)\right)=\min$$

### 2.3. Evaluation Metrics

This study uses the RMSE of the retrieval results to evaluate the performance of different TES algorithms on the inversion of the simulated radiances with different spectral resolutions. The RMSE of a group of retrieval LSTs with a certain spectral response setting and noise level is defined as:

(8) $${\mathrm{RMSE}}_{T}=\sqrt{\frac{1}{N_{s}}\sum_{i=1}^{N_{s}}{\left(T_{ri}-T_{oi}\right)}^{2}}$$

where $T_{ri}$ and $T_{oi}$ are the retrieval and original temperature respectively, $i\in \left[1,N_{s}\right]$, and $N_{s}$ is the number of samples (1525*15*6) in the case of a certain spectral response setting (a center wavelength shift and a FWHM change) and noise level.

The RMSE of a retrieval LSE is defined as:

(9) $${\mathrm{RMSE}}_{\varepsilon }=\sqrt{\frac{1}{N}\sum_{i=1}^{N}{\left(\varepsilon_{r}\left(\lambda_{i}\right)-\varepsilon_{o}\left(\lambda_{i}\right)\right)}^{2}}$$

where $\varepsilon_{r}\left(\lambda_{i}\right)$ and $\varepsilon_{o}\left(\lambda_{i}\right)$ are the retrieval and original emissivity at the *i*-th band respectively,$i\in \left[1,N\right]$, and *N* is the number of bands of an emissivity curve. The RMSE for a group of retrieval LSEs with a certain spectral response setting and noise level is the mean of the RMSEs of all the emissivity curves in the group.

## 3. Results

### 3.1. The Overall Distribution Trend of Inversion Errors

The study displayed the overall distribution trend of inversion errors with the center wavelength shift ratios, FWHM change ratios and instrument noise levels, in order to visualize the comprehensive influence of the three factors on inversion errors from a global perspective. It was found that the results from ISSTES perform differently with those from ARTEMISS, then the performance of the two algorithms was analyzed in the section.

**Figure 3:**
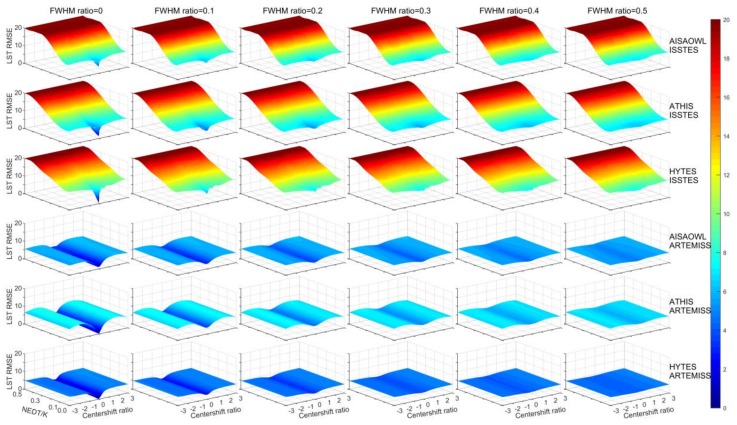
Distribution trend of the retrieval LST errors with the center wavelength shift ratios, FWHM change ratios and NEDTs. The sub-images have a same coordinate system with consistent axis ranges and color bar, where the X axis on the right hand side is the center wavelength shift ratio, the Y axis on the left hand side is instrument noise NEDT (K), the vertical Z axis is the LST RMSE (K), and the color is proportional to the error. Each sub-image has a constant the FWHM change ratio. 1–3 rows: LST errors of AISAOWL (100nm), ATHIS (50nm) and HYTES (35 nm) inversed by ISSTES respectively; 4–6 rows: LST errors of AISAOWL (100nm), ATHIS (50nm) and HYTES (35 nm) inversed by ARTEMISS respectively.

**Figure 4:**
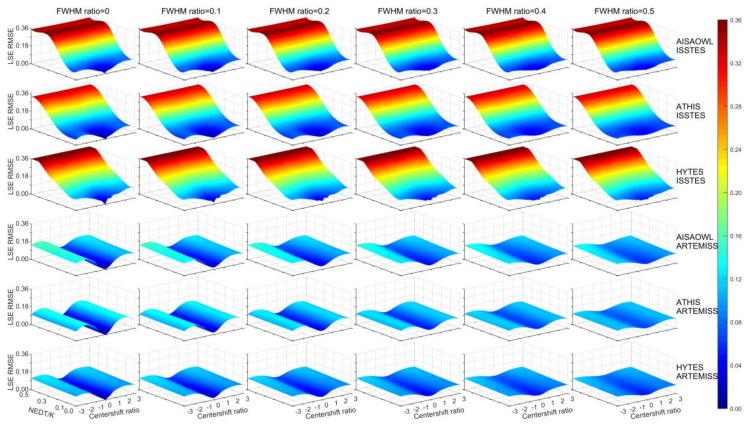
Distribution trend of the retrieval LSE errors with the center wavelength shift ratios, FWHM change ratios and NEDTs. The sub-images have a same coordinate system with consistent axis ranges and color bar, where the X axis on the right hand side is the center wavelength shift ratio, the Y axis on the left hand side is instrument noise NEDT (K), the Z axis is the LSE RMSE, and the color is proportional to the error. Each sub-image has a constant the FWHM change ratio. 1–3 rows: LSE errors of AISAOWL (100 nm), ATHIS (50 nm) and HYTES (35 nm) inversed by ISSTES respectively; 4–6 rows: LSE errors of AISAOWL (100 nm), ATHIS (50 nm) and HYTES (35 nm) inversed by ARTEMISS respectively.

Figure 3 and Figure 4 show the distribution trend of the retrieval LST and LSE errors obtained from the experiment, respectively, with the center wavelength shift ratios, FWHM change ratios and instrument noise levels. Overall, for each of the six groups of data, the relationship of retrieval LST errors with the three factors in Figure 3 shows the similar morphology and distribution with that of the retrieval LSE errors in Figure 4. 

For LST retrieval errors, taking the first row of Figure 3 as an example, each of the six sub-images shows the LST retrieval errors on 17 center wavelength shift ratios, six NEDTs and one FWHM change ratio for AISAOWL (100 nm) inversed by ISSTES. In one sub-image, the trend of LST errors with the center wavelength shift ratios and NEDTs is like a V-shaped valley with a sloping bottom, where the valley extends to both sides as the center wavelength shift ratio increases with 0 as the center and reaches its top as the central wavelength shift ratio reaches ±1.0 for each NEDT, while the bottom of the valley gradually rises and the width of the opening gradually decreases as NEDT increases. For FWHM change ratios shown in the six sub-images horizontally, the LST errors increase slightly with FWHM change ratios, where the bottom of the valley gradually rises as FWHM change ratio increases. Morphologically, Figure 3 shows the consistent trends of the LST retrieval errors with the three factors from different HTIR simulated data with different spectral resolutions inversed by different inversion methods, except that the V-shaped valley of ISSTES becomes flat when NEDT is greater than about 0.2 K while that of ARTEMISS exists for all six NEDTs. In terms of the order of magnitude of the LST retrieval errors, however, the ones from the same inversion method are similar to each other (e.g., 1st–3rd rows, 4th–6th rows), but there is big difference between the ones from different inversion methods, where the LST retrieval errors from ARTEMISS are much smaller than those from ISSTES.

There are lots of LST retrieval errors of about 20 K (shown in deep red color in Figure 3) among the results of ISSTES, which is supposed to be related to the temperature inversion strategy used in the study or the inversion algorithm itself. In order to reduce the computation time by reducing the computation complexity and avoid the local minimum value issue usually occurring with iterative strategies, the optimum search method based on a temperature range is used to search the optimal temperature of an object during the TES process. The cost functions are calculated for all the temperatures in the preset range, then the smallest cost function is found where the corresponding temperature will be considered as the optimal temperature. The temperature of one object is a physical parameter within a finite value range, although it is unknown before measured or estimated. For example, the temperatures of most ground objects on earth are in the range from −100 to 200 °C which can be used in the retrieval of the real earth observation data. Considering that the real temperature of one object to be inversed is known in the simulation and inversion experiments of the study, the temperature research range is set to $T+\Delta T_{s}$ (T is the real object temperature, $\Delta T_{s}$ is set to [−20K, 20K] in the study), which can further save computation time.

**Figure 5:**
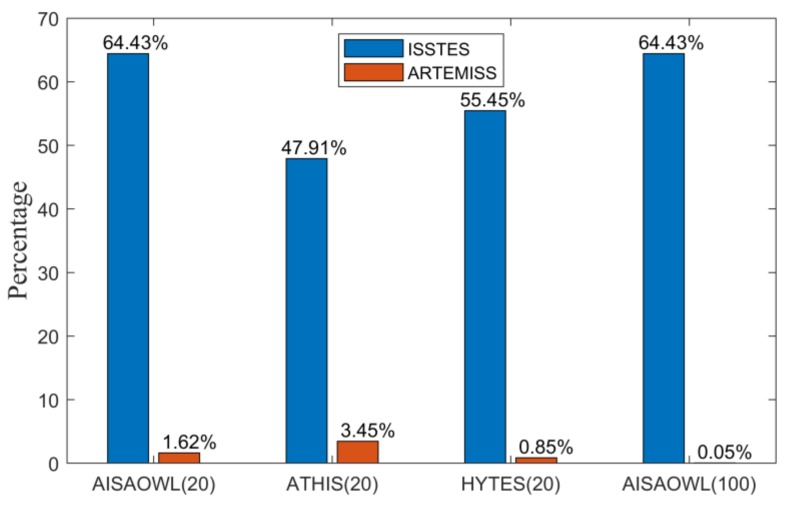
Percentage of the retrieval temperature error saturation phenomenon. The first three groups of bars are the percentages of the cases that the absolute value of retrieval LST error is 20 K in the retrieval results of ISSTES and ARTEMISS on AISAOWL (100 nm), ATHIS (50 nm) and HYTES (35 nm) data, respectively, when $\Delta T_{s}$ is set to [−20K, 20K]; the fourth group of bars are the percentages of the cases that the absolute value of retrieval LST error is 100 K in the retrieval results of the two algorithms on AISAOWL (100 nm) data respectively when $\Delta T_{s}$ is set to [−100 K, 100 K].

When the theoretical optimal temperature of an object that can be obtained by an inversion method is out of the temperature search range, its corresponding retrieval temperature will reach the upper or low limit of the search range where the cost function is the smallest. We call the case the retrieval temperature error saturation phenomenon, or an inversion failure for it is meaningless in the practical inversion application. We calculate the percentage of the error saturation cases in four groups of inversion experiments, shown in Figure 5 where the first three groups are AISAOWL, ATHIS and HYTES inversion experiments respectively with $\Delta T_{s}$ set to [−20K, 20K] and the fourth group is AISAOWL inversion experiment with $\Delta T_{s}$ set to [−100K, 100K]. From the first and fourth group of bars in Figure 5, it can be seen that for the same group of data, namely AISAOWL simulated data, the ratio of the retrieval temperature error saturation phenomenon when $\Delta T_{s}$ is set to [−100K, 100K] is nearly same as that when $\Delta T_{s}$ is set to [−20K, 20K]. Therefore, it can be inferred that the retrieval temperature error saturation phenomenon is not caused by the temperature search range being set too small, but may be caused by the limitation of the inversion algorithm itself (this point has little to do with the research content of this article, so we will not discuss it in detail here). Moreover, if the temperature search range is doubled, the running time of the inversion algorithm will also be doubled, however it improves no significant performance for the algorithm. So the temperature search range is not necessarily as large as possible. Concerning the algorithm performance and the time cost, we consider it reasonable to set $\Delta T_{s}$ to [−20K, 20K].

**Figure 6:**
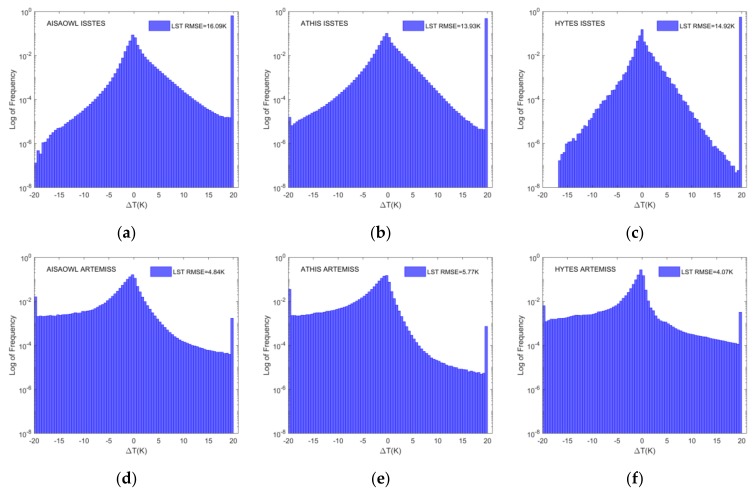
The overall LST error histogram. The X axis is the temperature error, the Y axis is the logarithm of frequency distribution of all retrieval LST errors with RMSE. (**a**–**c**) LST error histograms of AISAOWL (100 nm), ATHIS (50 nm) and HYTES (35 nm) inversed by ISSTES respectively; (**d**–**f**) LST error histograms of AISAOWL (100 nm), ATHIS (50 nm) and HYTES (35 nm) inversed by ARTEMISS respectively.

**Figure 7:**
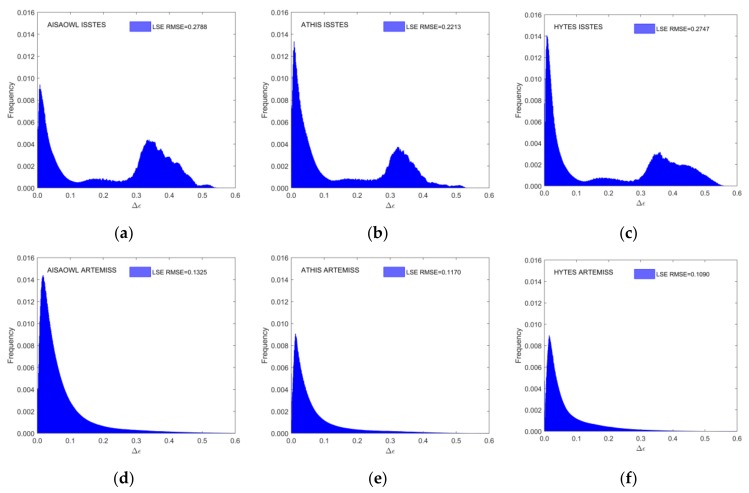
The overall LSE error histogram. The X axis is the LSE error, the right Y axis is the frequency distribution of all retrieval LSE errors and scales the red histogram with RMSE, and the left Y axis is the frequency distribution of the corresponding retrieval LSE errors excluding LST errors of ± 20K and scales the blue histogram in order to highlight the frequency distribution of the LSE error near 0. (**a**–**c**) LSE error histograms of AISAOWL (100 nm), ATHIS (50 nm) and HYTES (35 nm) inversed by ISSTES respectively; (**d**–**f**) LSE error histograms of AISAOWL (100 nm), ATHIS (50nm) and HYTES (35 nm) inversed by ARTEMISS respectively.

For intuitively evaluating the performances of ISSTES and ARTEMISS on three groups of HTIR data with three spectral resolutions, the study draws the histograms for the LST and LSE absolute errors (the retrieval LST or LSE minus the real LST or LSE), shown in Figure 6 and Figure 7, for AISAOWL (100 nm), ATHIS (50 nm) and HYTES (35 nm) simulated data inversed by the two TES algorithms.

Figure 6a–c show that a substantial part of the retrieval LSTs of ISSTES method have a maximum boundary temperature error of ±20 K, the retrieval LST errors excluding ±20 K are approximately normally distributed with 0 K as the center, and the RMSE of overall retrieval LST errors of AISAOWL, ATHIS, and HYTES simulated data are 16.09 K, 13.93 K, and 14.92 K, respectively. Figure 6d–f show that the proportions of LST errors with the maximum boundary error of ±20 K in the retrieval results of AISAOWL, ATHIS, and HYTES by ARTEMISS method is much less than those by ISSTES, the overall retrieval LST errors are approximately normally distributed with 0 K as the center too but more LST errors are negative than positive, and the RMSE of overall retrieval LST errors of AISAOWL, ATHIS, and HYTES are 4.84 K, 5.77 K, and 4.07 K, respectively. In the retrieval results of both ISSTES and ARTEMISS, the frequency of temperature error near 0K is less than 0.1.

Figure 7a–c show that due to the existence of many inversion failure cases, there are two peaks in the frequency distribution charts of the retrieval LSE errors resulted from ISSTES for AISAOWL, ATHIS and HYTES simulated data. One is between 0−0.05 and the other is between 0.3−0.4. Obviously, the latter is caused by those inversion failure cases. The RMSE of overall retrieval LSE errors inversed by ISSTES on AISAOWL, ATHIS, and HYTES are 0.2788, 0.2213 and 0.2747 respectively. Figure 7d–f show that the frequency distributions of the retrieval LSE errors resulted from ARTEMISS for AISAOWL, ATHIS and HYTES are unimodal, and their RMSE are 0.1325, 0.1170 and 0.1090, respectively.

The above results suggest that neither of the two TES methods can effectively overcome the effects of spectral calibration uncertainty and instrument noise. In terms of the proportion of inversion failures and the overall distribution of errors, the ARTEMISS method performs much better than the ISSTES method.

### 3.2. The Single-Factor Analysis for Inversion Errors

In order to study the separate influence of one of the instrument’s three impact factors (center wavelength shift ratio, FWHM change ratio and instrument noise) on the LST or LSE error, this paper analyses the relationship between the retrieval LST or LSE error and each of the three impact factors for the inversion results of the AISAOWL (100 nm), ATHIS (50 nm), and HYTES (35 nm) simulated data by ISSTES or ARTEMISS, respectively. In single-factor analysis, we draw the plots of the LST or LSE error (shown in the graphs in Figure 3 and Figure 4) on the third factor by averaging over the other two dimensions of the three factors. The following subsections will show the results of three factors respectively.

#### 3.2.1. The Relationship between Inversion Errors and Center Wavelength Shift Ratio

**Figure 8:**
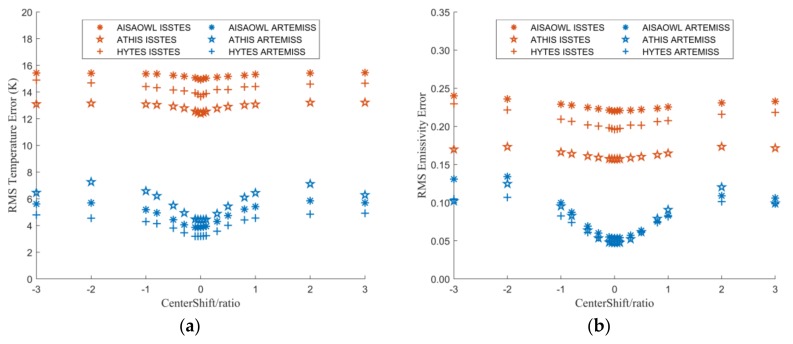
The graph of LST or LSE errors varying with center wavelength shift ratio. The X axis is the center wavelength shift ratio, and the Y axis is the LST error in (**a**) and is the LSE error in (**b**).

Figure 8 is a graph of the LST and LSE errors varying with center wavelength shift ratio. It shows that both the LST and LSE errors are basically centered on 0 and increase as the center wavelength shift ratio away from 0. The error trends between different sensors are relatively close, but the error trends of different TES methods are quite different. The errors of ISSTES are several times those of ARTEMISS. It suggests that when the spectral resolution of the HTIR data is on the order of tens of nanometers, the inversion method has a greater impact on the inversion results than the spectral resolution.

**Figure 9:**
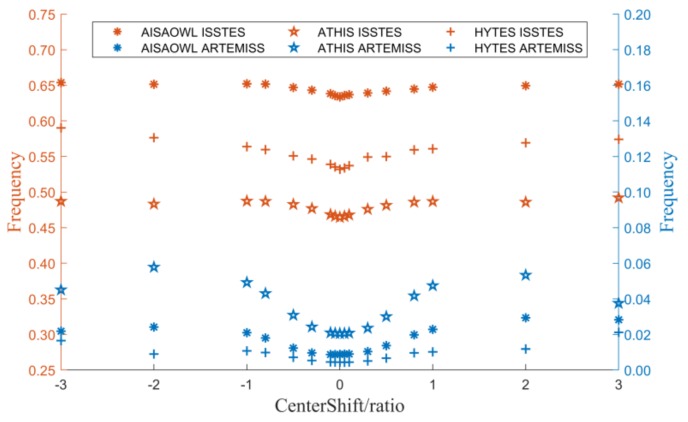
The frequency of inversion failure varying with the center wavelength shift ratio. The X axis is the center wavelength shift ratio, the left and right Y axis scale the results of ISSTES and ARTEMISS respectively.

There is a special phenomenon that when the center wavelength shift ratio is extended from ±1 to ±3, the errors of LST and LSE remain nearly the same, even decrease in some cases of ARTEMISS when the center wavelength shift is equal to 3. This may be due to the rapid increase in the percentage of inversion failures when the central wavelength shift is beyond ±1. In the cases, the proportion of inversion failures determine the average error at a certain center wavelength shift ratio. The frequency of inversion failures shown in Figure 9 is highly consistent with the trend of the errors in Figure 8. Furthermore, with the improvement of equipment development technology, current equipment’s center wavelength shift ratio rarely exceeds ±1. Therefore, in order to quantitatively describe the influence of the center wavelength shift ratio on the LST and LSE inversion errors, we fit the points in Figure 8 within the range of the center wavelength shift ratio of [−1,1], and obtain the fitting functions shown in Table 3 and Table 4. In terms of the fitting functions, the LST and LSE errors both increase rapidly from 0 to both sides as a quadratic function as the center wavelength shift ratio expands to both sides when the center wavelength shift is within the range of [−1,1].

#### 3.2.2. The Relationship between Inversion Errors and FWHM Change Ratio

**Figure 10:**
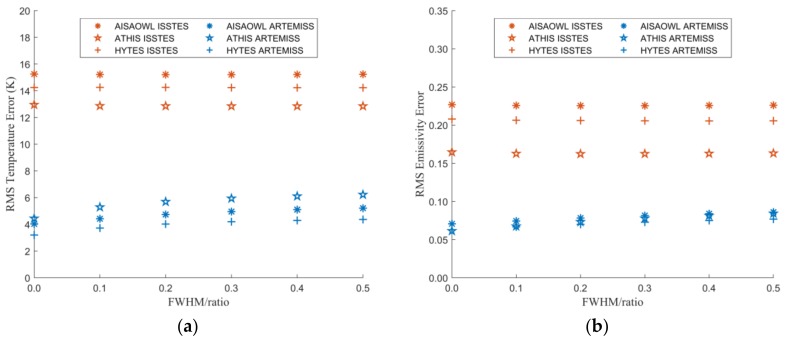
The graph of LST or LSE errors varying with the FWHM change ratio. The X axis is the FWHM change ratio, and the Y axis is LST error in (**a**) and is LSE error in (**b**).

Figure 10 is the graph of the LST and LSE errors versus FWHM change ratio. It shows that as the FWHM change ratio increases, both the LST and LSE errors appear to increase slightly. The error trends of different sensors with same TES method are relatively close, but the error trends of the two inversion methods are quite different. The inversion errors of ARTEMISS for the simulated data of different sensors are almost the same. Coinciding with the effect of the center wavelength shift ratio on the errors, the errors of ISSTES are several times those of ARTEMISS.

In order to quantitatively describe the influence of FWHM change ratio on the LST and LSE inversion errors, we fit the points in Figure 10 and obtain the fitting functions shown in Table 5 and Table 6. In terms of the fitting functions, the errors increase linearly with FWHM change ratio. However, the slopes of all the linear fitting functions are small. It implies that the change of FWHM has little effect on inversion errors.

#### 3.2.3. The Relationship between Inversion Errors and NEDT

**Figure 11:**
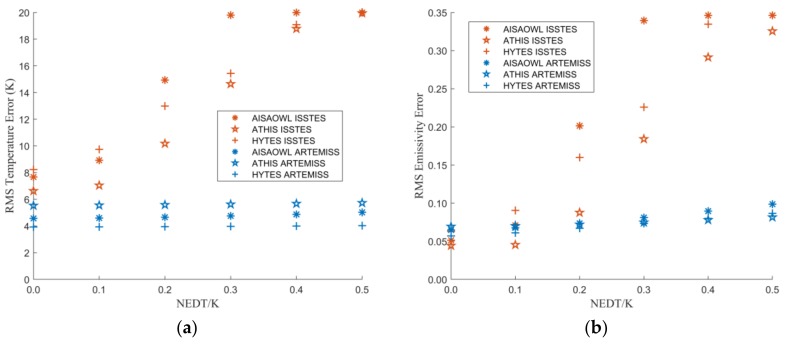
The graph of LST or LSE errors varying with NEDT. The X axis is NEDT, and the Y axis is LST error in (**a**) and is LSE error in (**b**).

Figure 11 is the graph of t the LST and LSE errors varying with NEDT. It shows that with the increase of instruments noise, the errors of the two TES methods show a different trend. Coinciding with the effect of FWHM change ratio and center wavelength shift ratio on the inversion errors, the error trends of different sensors are closer, while those of different TES methods are quite different, and the errors of ISSTES are several times those of ARTEMISS. As NEDT increases, the errors of ISSTES increase in an S-curve, while the errors of ARTEMISS increase slightly and linearly.

In order to quantitatively describe the influence of NEDT on the LST and LSE inversion errors, we fit the points in Figure 11 and obtain the fitting functions shown in Table 7 and Table 8. From the expression of the fitting functions, as the increase of the noise, the ISSTES inversion errors increase in an S-curve, while the ARTEMISS inversion error increases linearly. However, the slopes of all ARTEMISS linear fitting functions are small, which implies that ARTEMISS method has some degree of noise immunity, which agrees with the result of a previous research [23].

From the analyses above, it can be concluded that the center wavelength shift has a greater effect on the inversion errors, but the FWHM change has little effect on the inversion errors. Generally, the center wavelength shift can be corrected by the scene-based spectral calibration algorithm using the atmospheric absorption channel as a reference, and the minimum error is less than 1 nm under the influence of atmospheric conditions (such as atmospheric water vapor content) [40]. Here we assume that the scene-based spectral shift correction error is less than 0.05 band and analyze the characteristics of LST and LSE inversion errors after the spectral shift is corrected.

**Figure 12:**
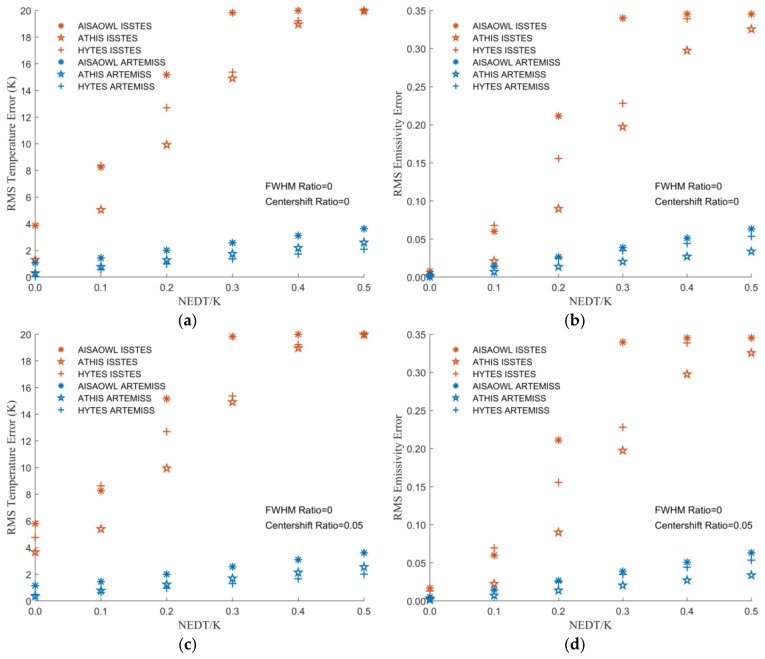

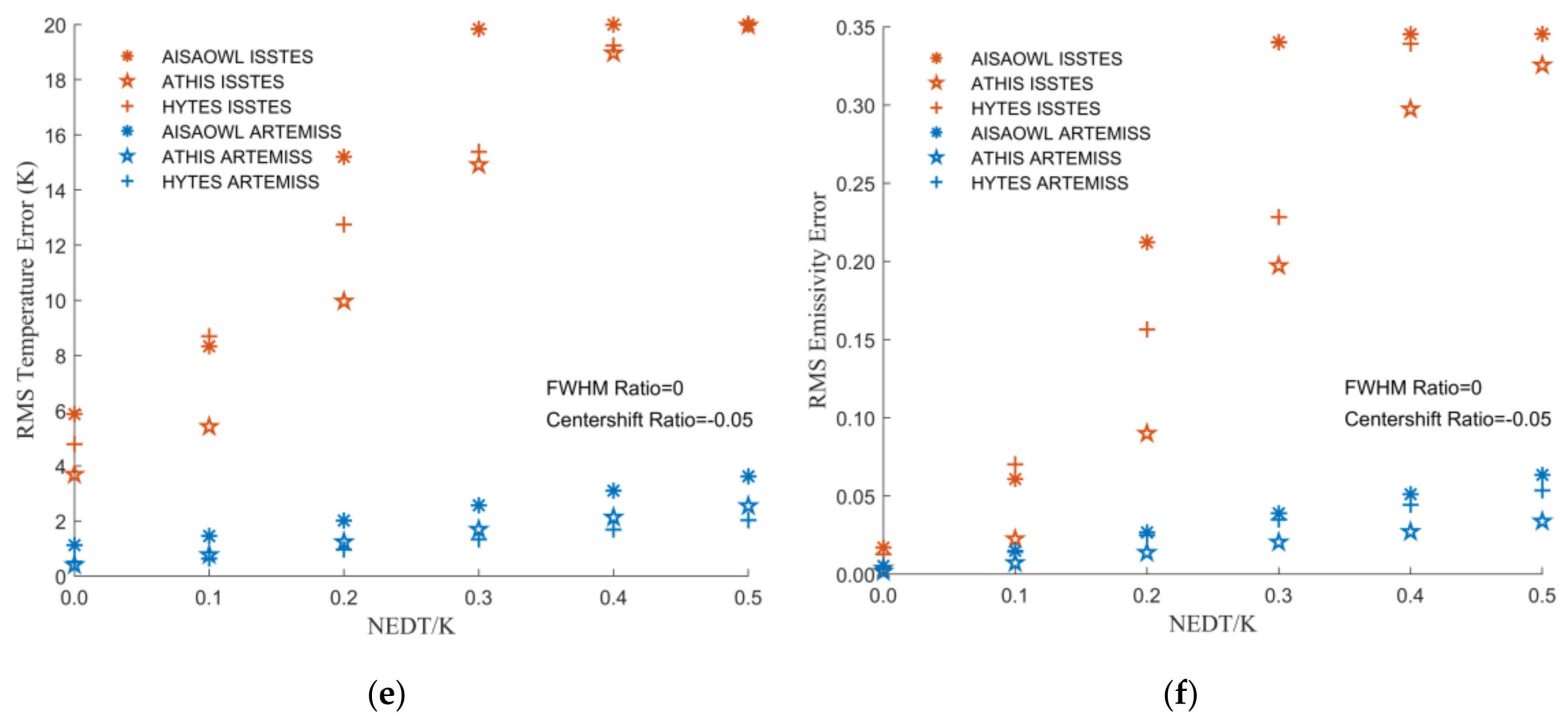
The graph of LST or LSE errors varying with NEDT when center wavelength shift ratio is in [−0.05, 0, 0.05] and FWHM change ratio is 0. The X axis is NEDT, and the Y axis is the LST error in (**a**,**c**,**e**)and is the LSE error in (**b**,**d**,**f**). (**a**,**b**) Center wavelength shift ratio is 0. (**c**,**d**) Center wavelength shift ratio is 0.05. (**e**,**f**) Center wavelength shift ratio is −0.05.

Figure 12 is a scatter plot of LST and LSE inversion errors varying with NEDT when the center wavelength shift ratio extracted from Figure 3 and Figure 4 is in [−0.05, 0, 0.05] and the FWHM change is 0. Table 9, Table 10, Table 11, Table 12, Table 13 and Table 14 show the optimal functions obtained by fitting the data in Figure 12. These charts show that the performance of ISSTES is much worse than ARTEMISS. When the NEDT exceeds 0.3 K, the inversions of ISSTES are almost entirely failed, while there are few failure cases in the inversions of ARTEMISS. When both the center wavelength shift ratio and NEDT are 0, the LST and LSE inversion errors of ISSTES and ARTEMISS, except the LST error of AISAOWL inversed by ISSTES, are almost the same. With the increase of the center wavelength shift ratio and NEDT, the errors of ISSTES increase rapidly and exponentially, while the errors of ARTEMISS only increase slowly and linearly with a small base.

From the perspective of fitting functions of the LST errors of ARTEMISS, the order of the intercepts of the functions is AISAOWL < ATHIS < HYTES. It seems that the smaller the NEDT and the higher the spectral resolution, the smaller the LST inversion error. As the NEDT increases, the LST inversion errors with a higher spectral resolution increase faster. However, there is no clear evidence that the relationship between the LSE errors and the spectral resolution is regular.

Through comprehensive observation of the fitting functions of the LST and LSE errors when center wavelength shift ratio is within [−0.05, 0, 0.05] and FWHM change ratio is 0, we find that when NEDT is 0.1 K, the LST errors of the instruments except AISAOWL are within 1 K; the LSE errors of all the three instruments are within 0.015. When the LST errors of the ATHIS and HYTES is within 0.5 K, NEDT is 0.03K where LSE error is within 0.01. Therefore, for meeting the requirements of some basic applications (LST error < 1 K) [20,41,42,43], the instrument’s NEDT should be designed at least within 0.1 K; for further improving the effect and level of the applications (LST error < 0.5 K), the instrument’s NEDT should be designed within 0.03 K.

## 4. Discussion

The research in this paper focused on the influence of the core performance indicators, in terms of spectra and noise, of the common HTIR instruments on the LST and LSE inversion errors. It provided valuable references for the performance improvement and data processing of HTIR instruments. However, further research is needed on the relationship between the inversion errors and the performance of the HTIR instruments with higher spectral resolution and the validation of the modelled effects with real thermal infrared hyperspectral data. Moreover, during the result analysis, we found that ISSTES method has its own limitations. It is intelligible that after all, every TES method has certain assumptions. This also results in some TES methods (for example, ISSTES) being strong sensitive to data distortion while some TES methods (for example, ARTEMISS) being weakly sensitive to data distortion. Therefore, while committed to improving the performance of the instrument, we should also pay attention to how to further improve the anti-data-distortion ability of the TES method, especially noise. Instrument noise cannot be avoided or precisely corrected with the data itself, while center wavelength shift and FWHM change can usually be corrected by some methods to reduce the errors. Besides, atmospheric correction error is also affected by factors such as center wavelength shift, FWHM change and NEDT, which in turn affects the error of the TES algorithms. It needs further research, especially, whether the superposition effect will amplify the influence of instrument spectral parameter changes and noise on the TES algorithms.

## 5. Conclusions

From the perspective of instrument development, this article took the development of the instrument as the requirement, selected three instruments with common spectral resolutions, two TES methods and ASTER 2.0 spectral library as the representative data, and analyzed the influence of the core performance indicators, in terms of spectral and noise, of the instrument on the LST and LSE inversion errors. After simulation, inversion and result analysis, the following conclusions are reached:

(1) For instruments with the spectral resolution of tens of nanometers, the difference in the instrument spectral resolutions has a small effect on the inversion errors, but the error trends of different inversion methods are quite different. The errors of ISSTES are several times those of ARTEMISS. ARTEMISS performs much better than ISSTES.
(2) Considering only the center wavelength shift, the LST and LSE inversion errors increase with center wavelength shift ratio in a quadratic function. When the center wavelength shift ratio exceeds ±1, the proportion of inversion failures rapidly increases. Therefore, it is highly recommended to keep the center wavelength shift ratio within ±1 as much as possible during instrument design and development, even if you may correct the center wavelength shift relying on the data after data acquisition.
(3) Compared with the center wavelength shift and instrument noise, FWHM change has the least influence on the inversion error among the three factors. The errors of the two inversion methods on three groups of simulated data with different spectral resolutions increase slowly and linearly with the increase of FWHM change ratio, and the slopes of all linear fitting functions are relatively small.
(4) The influences of instrument noise on the errors of different inversion methods behave differently. The errors of ISSTES increase with NEDT in an S-curve, while the errors of ARTEMISS increase with NEDT slightly and linearly.
(5) After the center wavelength shift of some data can be corrected by a method using the atmospheric absorption channel as a reference and the correction error is within 0.05 bands, if we use ARTEMISS method to inverse the corrected data, we will obtain a smaller inversion error. The smaller the NEDT and the higher the spectral resolution, the smaller the LST inversion errors of ARTEMISS. With the increase of NEDT, the LST inversion errors with high spectral resolution are growing fast. The instrument whose NEDT is within 0.1 K can meet the basic application (LST error < 1 K), and that whose NEDT within 0.03 K can further improve the application effect and level (LST error < 0.5 K).

## Figures and Tables

**Table 1:** Part of the input parameters of MODTRAN.

Parameter	Value	Description
MODEL	1	Tropical Atmosphere (15° North Latitude, 299.7 K)
	2	Mid-Latitude Summer (45° North Latitude, 294.2 K)
	3	Mid-Latitude Winter (45° North Latitude, 272.2 K)
	4	Sub-Arctic Summer (60° North Latitude, 287.2 K)
	5	Sub-Arctic Winter (60° North Latitude, 257.2 K)
H1ALT	2	Low Aerial Sensor Altitude/km
	10	High Aerial Sensor Altitude/km
	750	Spaceborne Sensor Altitude/km

**Table 2:** Parameters of the considered hyperspectral thermal infrared imagers.

Instrument	First Deployed	Spectral Range /μm	Spectral Resolution (FWHM)	Bands	NEDT/K	FOV/°
AisaOWL	2014	7.7–12.3	100 nm	96	0.2	24
ATHIS	2017	8.0–12.5	50 nm	181	0.17	40
HyTES	2013	7.5–12.0	35.2 nm	256	0.2	50

**Table 3:** The LST error fitting function about center wavelength shift ratio.

Simulated Data and TES Method	Error Fitting Function	RMSE(K)
AISAOWL ISSTES	$e_{11,}{}_{t\_\mathrm{rmse}}=\left\{\begin{array}{l} -0.387r_{\delta \lambda_{i}}{}^{2}-0.7881r_{\delta \lambda_{i}}+14.97,r_{\delta \lambda_{i}}<0\\ -0.1851r_{\delta \lambda_{i}}{}^{2}+0.535r_{\delta \lambda_{i}}+14.96,r_{\delta \lambda_{i}}\geq 0 \end{array}\right.$	0.02
ATHIS ISSTES	$e_{21,}{}_{t\_\mathrm{rmse}}=\left\{\begin{array}{l} -0.718r_{\delta \lambda_{i}}{}^{2}-1.368\delta \lambda_{i}+12.43,r_{\delta \lambda_{i}}<0\\ -0.7059\delta \lambda_{i}{}^{2}+1.363\delta \lambda_{i}+12.41,r_{\delta \lambda_{i}}\geq 0 \end{array}\right.$	0.02
HYTES ISSTES	$e_{31,}{}_{t\_\mathrm{rmse}}=\left\{\begin{array}{l} -0.2974r_{\delta \lambda_{i}}{}^{2}-0.8846r_{\delta \lambda_{i}}+13.82,r_{\delta \lambda_{i}}<0\\ -0.8403r_{\delta \lambda_{i}}{}^{2}+1.519r_{\delta \lambda_{i}}+13.72,r_{\delta \lambda_{i}}\geq 0 \end{array}\right.$	0.05
AISAOWL ARTEMIISS	$e_{12,}{}_{t\_\mathrm{rmse}}=\left\{\begin{array}{l} 0.3079r_{\delta \lambda_{i}}{}^{2}-1.165r_{\delta \lambda_{i}}+3.757,r_{\delta \lambda_{i}}<0\\ -0.3319r_{\delta \lambda_{i}}{}^{2}+1.984r_{\delta \lambda_{i}}+3.802,r_{\delta \lambda_{i}}\geq 0 \end{array}\right.$	0.06
ATHIS ARTEMIISS	$e_{22,}{}_{t\_\mathrm{rmse}}=\left\{\begin{array}{l} -0.2088r_{\delta \lambda_{i}}{}^{2}-2.573r_{\delta \lambda_{i}}+4.249,r_{\delta \lambda_{i}}<0\\ -0.0864r_{\delta \lambda_{i}}{}^{2}+2.076r_{\delta \lambda_{i}}+4.328,r_{\delta \lambda_{i}}\geq 0 \end{array}\right.$	0.06
HYTES ARTEMIISS	$e_{32,}{}_{t\_\mathrm{rmse}}=\left\{\begin{array}{l} -0.337r_{\delta \lambda_{i}}{}^{2}-1.576r_{\delta \lambda_{i}}+3.075,r_{\delta \lambda_{i}}<0\\ -0.3902r_{\delta \lambda_{i}}{}^{2}+1.87r_{\delta \lambda_{i}}+3.12,r_{\delta \lambda_{i}}\geq 0 \end{array}\right.$	0.05

**Table 4:** The LSE error fitting function about center wavelength shift ratio.

Simulated Data and TES Method	Error Fitting Function	RMSE
AISAOWL ISSTES	$e_{11,}{}_{e\_\mathrm{rmse}}=\left\{\begin{array}{l} -0.0013r_{\delta \lambda_{i}}{}^{2}-0.0103r_{\delta \lambda_{i}}+0.2202,r_{\delta \lambda_{i}}<0\\ 0.0025r_{\delta \lambda_{i}}{}^{2}+0.0025r_{\delta \lambda_{i}}+0.2202,r_{\delta \lambda_{i}}\geq 0 \end{array}\right.$	2.305 × 10^−4^
ATHIS ISSTES	$e_{21,e\_\mathrm{rmse}}=\left\{\begin{array}{l} 0.0017r_{\delta \lambda_{i}}{}^{2}-0.0076r_{\delta \lambda_{i}}+0.1568,r_{\delta \lambda_{i}}<0\\ 0.0022r_{\delta \lambda_{i}}{}^{2}+0.0057r_{\delta \lambda_{i}}+0.1568,r_{\delta \lambda_{i}}\geq 0 \end{array}\right.$	8.276 × 10^−5^
HYTES ISSTES	$e_{31,e\_\mathrm{rmse}}=\left\{\begin{array}{l} 0.0008r_{\delta \lambda_{i}}{}^{2}-0.0122r_{\delta \lambda_{i}}+0.0196,r_{\delta \lambda_{i}}<0\\ -0.003r_{\delta \lambda_{i}}{}^{2}+0.0144r_{\delta \lambda_{i}}+0.0196,r_{\delta \lambda_{i}}\geq 0 \end{array}\right.$	7.565 × 10^−4^
AISAOWL ARTEMIISS	$e_{12,e\_\mathrm{rmse}}=\left\{\begin{array}{l} 0.0304r_{\delta \lambda_{i}}{}^{2}-0.0168r_{\delta \lambda_{i}}+0.0532,r_{\delta \lambda_{i}}<0\\ 0.0206r_{\delta \lambda_{i}}{}^{2}+0.0097r_{\delta \lambda_{i}}+0.0532,r_{\delta \lambda_{i}}\geq 0 \end{array}\right.$	8.011 × 10^−4^
ATHIS ARTEMIISS	$e_{22,e\_\mathrm{rmse}}=\left\{\begin{array}{l} 0.0305r_{\delta \lambda_{i}}{}^{2}-0.0197r_{\delta \lambda_{i}}+0.0461,r_{\delta \lambda_{i}}<0\\ 0.0299r_{\delta \lambda_{i}}{}^{2}+0.0155r_{\delta \lambda_{i}}+0.0461,r_{\delta \lambda_{i}}\geq 0 \end{array}\right.$	1.152 × 10^−3^
HYTES ARTEMIISS	$e_{32,}{}_{e\_\mathrm{rmse}}=\left\{\begin{array}{l} 0.0195r_{\delta \lambda_{i}}{}^{2}-0.0149r_{\delta \lambda_{i}}+0.0487,r_{\delta \lambda_{i}}<0\\ 0.0188r_{\delta \lambda_{i}}{}^{2}+0.0149r_{\delta \lambda_{i}}+0.0487,r_{\delta \lambda_{i}}\geq 0 \end{array}\right.$	8.751 × 10^−4^

**Table 5:** The LST error fitting function about FWHM change ratio.

Simulated Data and TES Method	Error Fitting Function	RMSE(K)
AISAOWL ISSTES	$e_{11,}{}_{t\_\mathrm{rmse}}=0.0558r_{\delta \Delta \lambda_{i}}+15.14$	0.02
ATHIS ISSTES	$e_{21,}{}_{t\_\mathrm{rmse}}=0.0211r_{\delta \Delta \lambda_{i}}+12.77$	0.03
HYTES ISSTES	$e_{31,t\_\mathrm{rmse}}=0.1372r_{\delta \Delta \lambda_{i}}+14.07$	0.02
AISAOWL ARTEMIISS	$e_{12,t\_\mathrm{rmse}}=3.128r_{\delta \Delta \lambda_{i}}+3.662$	0.14
ATHIS ARTEMIISS	$e_{22,t\_\mathrm{rmse}}=4.438r_{\delta \Delta \lambda_{i}}+4.147$	0.34
HYTES ARTEMIISS	$e_{32,t\_\mathrm{rmse}}=3.043r_{\delta \Delta \lambda_{i}}+2.957$	0.23

**Table 7:** The LST error fitting function about NEDT.

Simulated Data and TES Method	Error Fitting Function	RMSE(K)
AISAOWL ISSTES	$e_{11,}{}_{t\_\mathrm{rmse}}=1/\left(0.0684+1.637e^{-18.31\eta }\right)+6$	0.55
ATHIS ISSTES	$e_{21,}{}_{t\_\mathrm{rmse}}=1/\left(0.0686+3.429e^{-14.57\eta }\right)+5.923$	0.21
HYTES ISSTES	$e_{31,t\_\mathrm{rmse}}=1/\left(0.0645+0.527e^{-8.796\eta }\right)+6.095$	0.38
AISAOWL ARTEMIISS	$e_{12,t\_\mathrm{rmse}}=1.065\eta +4.178$	0.05
ATHIS ARTEMIISS	$e_{22,t\_\mathrm{rmse}}=0.4793\eta +5.137$	0.01
HYTES ARTEMIISS	$e_{32,t\_\mathrm{rmse}}=0.2301\eta +3.66$	0.01

**Table 8:** The LSE RMSE error fitting function about NEDT.

Simulated Data and TES Method	Error Fitting Function	RMSE
AISAOWL ISSTES	$e_{11,}{}_{e\_\mathrm{rmse}}=1/\left(3.3+1178e^{-29.83\eta }\right)+0.0448$	5.603 × 10^−3^
ATHIS ISSTES	$e_{21,}{}_{e\_\mathrm{rmse}}=1/\left(3.292+499.9e^{-16.86\eta }\right)+0.0323$	4.626 × 10^−3^
HYTES ISSTES	$e_{31,e\_\mathrm{rmse}}=1/\left(2.702+48.49e^{-10.34\eta }\right)+0.0331$	0.0132
AISAOWL ARTEMIISS	$e_{12,e\_\mathrm{rmse}}=0.0773\eta +0.0473$	2.344 × 10^−3^
ATHIS ARTEMIISS	$e_{22,e\_\mathrm{rmse}}=0.0286\eta +0.0557$	1.126 × 10^−3^
HYTES ARTEMIISS	$e_{32,e\_\mathrm{rmse}}=0.0661\eta +0.0444$	9.203 × 10^−4^

**Table 9:** The LST error fitting function about NEDT when center wavelength shift ratio = 0 and FWHM change ratio = 0.

Simulated Data and TES Method	Error Fitting Function	RMSE(K)
AISAOWL ISSTES	$e_{11,}{}_{t\_\mathrm{rmse}}=1/\left(0.0579+0.8993e^{-18.54\eta }\right)+3$	0.33
ATHIS ISSTES	$e_{21,}{}_{t\_\mathrm{rmse}}=1/\left(0.0479+0.5041e^{-11.19\eta }\right)$	0.35
HYTES ISSTES	$e_{31,t\_\mathrm{rmse}}=1/\left(0.0045+0.0005e^{-4.24\eta }\right)-199.3$	0.58
AISAOWL ARTEMIISS	$e_{12,t\_\mathrm{rmse}}=5.246\eta +1.002$	0.06
ATHIS ARTEMIISS	$e_{22,t\_\mathrm{rmse}}=4.641\eta +0.3268$	0.04
HYTES ARTEMIISS	$e_{32,t\_\mathrm{rmse}}=4.01\eta +0.1364$	0.06

**Table 10:** The LSE error fitting function about NEDT when center wavelength shift ratio = 0 and FWHM change ratio = 0.

Simulated Data and TES Method	Error Fitting Function	RMSE
AISAOWL ISSTES	$e_{11,}{}_{e\_\mathrm{rmse}}=1/\left(2.848+133.2e^{-21.67\eta }\right)$	9.035 × 10^−3^
ATHIS ISSTES	$e_{21,}{}_{e\_\mathrm{rmse}}=1/\left(2.953+172e^{-14.84\eta }\right)$	5.580 × 10^−3^
HYTES ISSTES	$e_{31,e\_\mathrm{rmse}}=1/\left(2.053+12.35e^{-7.877\eta }\right)-0.0662$	0.0152
AISAOWL ARTEMIISS	$e_{12,e\_\mathrm{rmse}}=0.1196\eta +0.0032$	5.725 × 10^−4^
ATHIS ARTEMIISS	$e_{22,e\_\mathrm{rmse}}=0.0658\eta +0.0007$	2.239 × 10^−4^
HYTES ARTEMIISS	$e_{32,e\_\mathrm{rmse}}=0.105\eta +0.0023$	1.634 × 10^−3^

**Table 11:** The LST error fitting function about NEDT when center wavelength shift ratio = 0.05 and FWHM change ratio = 0.

Simulated Data and TES Method	Error Fitting Function	RMSE(K)
AISAOWL ISSTES	$e_{11,}{}_{t\_\mathrm{rmse}}=1/\left(0.0681+2.875e^{-22.22\eta }\right)+5.51$	0.22
ATHIS ISSTES	$e_{21,}{}_{t\_\mathrm{rmse}}=1/\left(0.0555+1.053e^{-12.5\eta }\right)+2.72$	0.21
HYTES ISSTES	$e_{31,t\_\mathrm{rmse}}=1/\left(0.0459+0.1547e^{-7.504\eta }\right)$	0.46
AISAOWL ARTEMIISS	$e_{12,t\_\mathrm{rmse}}=5.083\eta +1.044$	0.07
ATHIS ARTEMIISS	$e_{22,t\_\mathrm{rmse}}=4.432\eta +0.3506$	0.01
HYTES ARTEMIISS	$e_{32,t\_\mathrm{rmse}}=3.168\eta +0.3831$	0.07

**Table 12:** The LSE error fitting function about NEDT when center wavelength shift ratio = 0.05 and FWHM change ratio = 0.

Simulated Data and TES Method	Error Fitting Function	RMSE
AISAOWL ISSTES	$e_{11,}{}_{e\_\mathrm{rmse}}=1/\left(3.003+261.6e^{-24.28\eta }\right)+0.0166$	8.898 × 10^−3^
ATHIS ISSTES	$e_{21,}{}_{e\_\mathrm{rmse}}=1/\left(3.003+262.2e^{-24.29\eta }\right)+0.0166$	8.898 × 10^−3^
HYTES ISSTES	$e_{31,e\_\mathrm{rmse}}=1/\left(2.251+18.64e^{-8.704\eta }\right)-0.0322$	0.0146
AISAOWL ARTEMIISS	$e_{12,e\_\mathrm{rmse}}=0.1173\eta +0.0039$	9.61 × 10^−4^
ATHIS ARTEMIISS	$e_{22,e\_\mathrm{rmse}}=0.0653\eta +0.001$	3.597 × 10^−4^
HYTES ARTEMIISS	$e_{32,e\_\mathrm{rmse}}=0.0995\eta +0.0044$	6.017 × 10^−4^

**Table 13:** The LST error fitting function about NEDT when center wavelength shift ratio = −0.05 and FWHM change ratio = 0.

Simulated Data and TES Method	Error Fitting Function	RMSE(K)
AISAOWL ISSTES	$e_{11,}{}_{t\_\mathrm{rmse}}=1/\left(0.0687+3.04e^{-22.45\eta }\right)+5.635$	0.35
ATHIS ISSTES	$e_{21,}{}_{t\_\mathrm{rmse}}=1/\left(0.0555+1.044e^{-12.45\eta }\right)+2.733$	0.33
HYTES ISSTES	$e_{31,t\_\mathrm{rmse}}=1/\left(0.046+0.153e^{-7.506\eta }\right)$	0.52
AISAOWL ARTEMIISS	$e_{12,t\_\mathrm{rmse}}=5.132\eta +1.029$	0.07
ATHIS ARTEMIISS	$e_{22,t\_\mathrm{rmse}}=4.354\eta +0.3748$	0.03
HYTES ARTEMIISS	$e_{32,t\_\mathrm{rmse}}=3.139\eta +0.4118$	0.08

**Table 14:** The LSE error fitting function about NEDT when center wavelength shift ratio = −0.05 and FWHM change ratio = 0.

Simulated Data and TES Method	Error Fitting Function	RMSE
AISAOWL ISSTES	$e_{11,}{}_{e\_\mathrm{rmse}}=1/\left(3.002+254.5e^{-24.21\eta }\right)+0.0166$	9.081 × 10^−3^
ATHIS ISSTES	$e_{21,}{}_{e\_\mathrm{rmse}}=1/\left(2.945+162.1e^{-14.62\eta }\right)$	4.79 × 10^−3^
HYTES ISSTES	$e_{31,e\_\mathrm{rmse}}=1/\left(2.183+16.2e^{-8.3\eta }\right)-0.04$	0.0113
AISAOWL ARTEMIISS	$e_{12,e\_\mathrm{rmse}}=0.1175\eta +0.0041$	9.654 × 10^−4^
ATHIS ARTEMIISS	$e_{22,e\_\mathrm{rmse}}=0.0648\eta +0.0012$	3.929 × 10^−4^
HYTES ARTEMIISS	$e_{32,e\_\mathrm{rmse}}=0.1\eta +0.0043$	6.212 × 10^−4^

**Table 6:** The LSE error fitting function about FWHM change ratio.

Simulated Data and TES Method	Error Fitting Function	RMSE
AISAOWL ISSTES	$e_{11,}{}_{e\_\mathrm{rmse}}=0.0005r_{\delta \Delta \lambda_{i}}+0.223$	6.216 × 10^−4^
ATHIS ISSTES	$e_{21,}{}_{e\_\mathrm{rmse}}=0.0005r_{\delta \Delta \lambda_{i}}+0.1601$	9.994 × 10^−4^
HYTES ISSTES	$e_{31,e\_\mathrm{rmse}}=-0.0009r_{\delta \Delta \lambda_{i}}+0.2018$	5.734 × 10^−4^
AISAOWL ARTEMIISS	$e_{12,e\_\mathrm{rmse}}=0.0497r_{\delta \Delta \lambda_{i}}+0.0542$	1.167 × 10^−3^
ATHIS ARTEMIISS	$e_{22,e\_\mathrm{rmse}}=0.0661r_{\delta \Delta \lambda_{i}}+0.0463$	1.771 × 10^−3^
HYTES ARTEMIISS	$e_{32,e\_\mathrm{rmse}}=0.042r_{\delta \Delta \lambda_{i}}+0.0504$	1.039 × 10^−3^
